# Molecular mechanism of SCARB2-mediated attachment and uncoating of EV71

**DOI:** 10.1007/s13238-014-0087-3

**Published:** 2014-07-02

**Authors:** Minghao Dang, Xiangxi Wang, Quan Wang, Yaxin Wang, Jianping Lin, Yuna Sun, Xuemei Li, Liguo Zhang, Zhiyong Lou, Junzhi Wang, Zihe Rao

**Affiliations:** 1 National Laboratory of Macromolecules, Institute of Biophysics, Chinese Academy of Science, Beijing, 100101 China; 2 Laboratory of Structural Biology, School of Medicine, Tsinghua University, Beijing, 100084 China; 3 School of Life Sciences, School of Pharmacy, Nankai University, Tianjin, 300071 China; 4 National Institutes for Food and Drug Control, Beijing, 100050 China

**Keywords:** viral entry, uncoating, picornaviruses, receptor binding, SCARB2, EV71, lipid transfer tunnel

## Abstract

Unlike the well-established picture for the entry of enveloped viruses, the mechanism of cellular entry of non-enveloped eukaryotic viruses remains largely mysterious. Picornaviruses are representative models for such viruses, and initiate this entry process by their functional receptors. Here we present the structural and functional studies of SCARB2, a functional receptor of the important human enterovirus 71 (EV71). SCARB2 is responsible for attachment as well as uncoating of EV71. Differences in the structures of SCARB2 under neutral and acidic conditions reveal that SCARB2 undergoes a pivotal pH-dependent conformational change which opens a lipid-transfer tunnel to mediate the expulsion of a hydrophobic pocket factor from the virion, a pre-requisite for uncoating. We have also identified the key residues essential for attachment to SCARB2, identifying the canyon region of EV71 as mediating the receptor interaction. Together these results provide a clear understanding of cellular attachment and initiation of uncoating for enteroviruses.

**Electronic supplementary material:**

The online version of this article (doi:10.1007/s13238-014-0087-3) contains supplementary material, which is available to authorized users.

## Introduction

Cell entry of non-enveloped viruses can be accomplished through two distinct pathways: (a) the endocytic pathway that employs clathrin-coated vesicles or caveolae; or (b) the formation of a pore on the cell surface (Hogle, 2002; Iwata et al., 1991; Marsh and Helenius, 2006; Smith and Helenius, 2004). Picornaviruses are typical non-enveloped viruses and their entry into the host cell is facilitated mainly through the endocytic pathway (Tuthill et al., 2010). The process of Picornavirus ingress can be subdivided into two key steps—(I) attachment, in which the virus binds to its functional receptors on the surface of the host cell; and (II) uncoating, in which the viral genome is released from viral particles into host cells (Bergelson and Coyne, 2013). Uncoating of many Picornaviruses requires expulsion of a natural lipid, also known as “the pocket factor”, from the viral capsid. Removal of the lipid triggers conformational changes of the capsid, leading to formation of uncoating intermediates called A particles (135 S) (Chen et al., 2012; Crowell and Philipson, 1971; Lonberg-Holm et al., 1975; Wang et al., 2012) that culminates in the release of the N-termini of VP1 (Fricks and Hogle, 1990; Ren et al., 2013) and all of VP4 (De Sena and Mandel, 1977). The viral genome is eventually released through the opening created by this partial disassembly of the capsid.

Enterovirus 71 (EV71) and Coxsackievirus A16 (CVA16) are representative members of the enterovirus (EV) genus of Picornaviruses and are two major causative agents of hand-foot-and-mouth disease (HFMD) in the Asia-Pacific region (Huang et al., 1999; Lum et al., 1998; Sun et al., 2013). To date, two human membrane proteins, P-selectin glycoprotein ligand-1 (PSGL-1 or CD162) and scavenger receptor class B 2 (SCARB2 or CD36-like-2), have been identified as functional receptors for EV71 (Nishimura et al., 2009; Yamayoshi et al., 2009). SCARB2 is ubiquitously expressed and serves as a common receptor for all clinically isolated EV71 strains, CVA16, CVA7 and CVA14 (Yamayoshi et al., 2012). Interestingly, SCARB2 was first identified as a receptor for β-glucocerebrosidase (β-GC) (Reczek et al., 2007) [loss-of-function mutations of β-GC result in Gaucher disease (GD) (Velayati et al., 2011)]. Lumenal acidification triggers the dissociation of β-GC from SCARB2 in late endosomal/lysosomal compartments in a pH-dependent manner (Zachos et al., 2012).

Some receptors not only play a role in the attachment of viruses to host cells, but also assist in viral uncoating (Arita et al., 1998; Cohen et al., 2001; Hogle, 2002; Tuthill et al., 2010). Unlike PSGL-1, SCARB2 is such an uncoating receptor for EV71 (Yamayoshi et al., 2013). Uncoating of EV71 is known to occur in endosomes and is tied to the progressive acidification of the endosome (Leong et al., 2011; Lin et al., 2012). Intriguingly, EV71 by itself is stable under acidic conditions (pH 4), and does not undergo any notable pH-dependent conformational changes (Plevka et al., 2012; Wang et al., 2012). However, SCARB2 has been shown to trigger the uncoating process of EV71 in an acidic environment (Chen et al., 2012; Yamayoshi et al., 2013). This raises possibility that EV71 uncoating is induced by conformational changes associated with SCARB2 at lower pH values. To answer this question and shed light on the molecular basis of SCARB2-mediated entry of EV71, structural, functional and biochemical studies were used to probe the receptor-virus interaction. We report here structures of SCARB2 under neutral and acidic pH, revealing pH-dependent conformational changes at viral binding domain and identify the binding interface between SCARB2 and EV71. Together with that SCARB2 can only dislodge natural lipids from EV71 virions and trigger viral uncoating at acidic conditions, these allow us to propose an evidence-based mechanism for receptor-mediated viral uncoating.

## Results

### Structures of SCARB2 under neutral and acidic pH conditions reveal a pivotal conformational change

**Figure 1:**
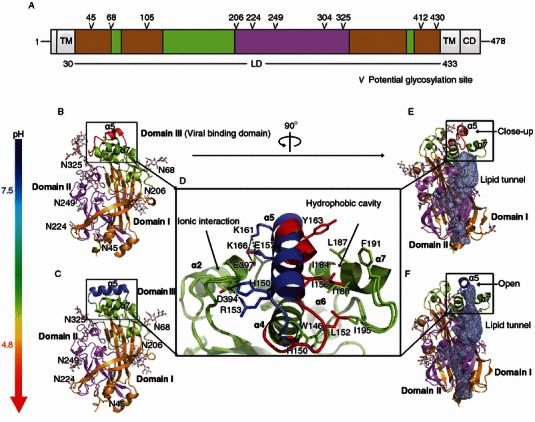
**Overview of the structures and characteristics of SCARB2**. (A) Schematic diagram of domain organization of SCARB2. TM, transmembrane domain; LD, luminal domain; CD, cytoplasmic domain. The overall structures of nSCARB2 (B) and aSCARB2 (C) are shown as cartoons. Domain I, II and III are colored in orange, violet and lemon, respectively. Glycans, cysteine residues and disulfide bonds are depicted as colored sticks. Helices α5 of nSCARB2 and aSCARB2 are highlighted in red and slate respectively. (D) Superposition of domain III of nSCARB2 and aSCARB2. The residues making ionic interactions and hydrophobic interactions are shown as colored sticks. The luminal tunnels of nSCARB2 (E) and aSCARB2 (F) were generated by HOLLOW (Ho and Gruswitz, 2008) software and are shown as blue mesh. See also Fig. S1 and Table S1

To gain an understanding of the molecular basis of SCARB2-mediated uncoating of EV71 under acidic conditions, we solved the crystal structures of the ectodomain of SCARB2 under both neutral (pH 7.5) (hereafter denoted as nSCARB2) and acidic conditions (pH 4.8) (hereafter denoted as aSCARB2) (Fig. 1 and Table S1). As expected, the overall structures of nSCARB2 and aSCARB2 are similar. SCARB2 (residues 37–430) consists of three domains arranged in a “Kidney” shape (Fig. 1B and 1C). Domain I comprises residues 37–69, 81–125, 334–390 and 403–429 and primarily contributes to a long twisted β-barrel structure at the core of the protein. Domain II, encompassing residues 206–333, contributes to the structural elements that complete the atypical β-barrel (Fig. 1B and 1C). A long tunnel that traverses the entire length of the protein from the membrane proximal end to the surface of the ectodomain passes through the hollow core of this β-barrel (Fig. 1E and 1F). Domain II harbors five glycosylation sites and two disulfide bridges (C274–C329 and C312–C318). Domain III is smaller and consists of residues 126–205, 70–80 and 391–402. Notably, a cluster of three helices (α4, α5 and α7) from this domain cap one end of the tunnel formed by domains I and II. Previous biochemical and mutagenesis studies suggest a role for amino acids from this “cap” in the interaction of SCARB2 with its ligands like β-GC and EV71 (Chen et al., 2012; Reczek et al., 2007).

The structure of aSCARB2 (pH 4.8) is similar to the recently published pH 5.5 structure [PDB code: 4F7B (Neculai et al., 2013)], with an r.m.s. deviation of 0.41 Å. Given the relatively low resolution of the present analysis (~3.7 Å, See Supplementary Information), this is at the level of experimental error and suggests that the specific features of the acidic structure observed are not artifacts of particular crystallization conditions. In contrast the elucidation of the structure of nSCARB2 (pH 7.5) at higher resolution (3.0 Å) and its comparison with aSCARB2 enabled us to visualize a pivotal conformational change in SCARB2 induced by pH that has implications for the function of the receptor. A striking difference in the position of the “cap” is observed between structures at acidic and neutral pH. In particular, helices α4 and α5, which are part of the β-GC binding site (residues 150–167) (Reczek et al., 2007) and most of the mapped EV71 binding site (residues 144–151) (Chen et al., 2012) assume different conformations.

### Transport through the lipid transfer tunnel of SCARB2 is regulated by pH

SCARB2 harbors a large cavity that is believed to serve as a route for transporting lipids in a way similar to the lipid-transfer tunnels of CD36 and SR-BI (Neculai et al., 2013). The structure of aSCARB2 reveals a penetrable tunnel, which traverses the entire length of the molecule (Figs. 1F and S1A). In contrast, at pH 7.5, the re-positioning of α5 in vicinity of α4 and α7 blocks the membrane-distal end of the tunnel, decreasing its void volume from 4,867 Å^3^ to 4,554 Å^3^ [calculated by program HOLLOW (Ho and Gruswitz, 2008)] (Figs. 1E,1F, S1A and S1B). Thus in nSCARB2, α5 (residues 157–164) adopts a short helical conformation. The “S” shaped linker (residues 148–155) connecting α4 with α5 is probably a key mediator of conformational changes. Helices α4, α5 and α7 are held together in a compact cluster by hydrophobic interactions involving residues W146, L152, I156, Y163 of α4–α5 and I184, L187, I188, F191, I195 from α7. In contrast, under acidic conditions, we propose that an alteration in the protonation state of H150 triggers the re-arrangement of the linker region connecting α4 with α5 such that the linker is now part of α5. Helix α4 is shortened in length, giving rise to a new linker (residues 146–150), that folds away from α7, connecting a much longer α5 with α4 (Fig. 1D). More importantly, α5 moves towards α15 and together with the conformational change of W146, extends the lipid-transfer tunnel right through to the membrane-distal end of the molecule with an open entrance. This movement is facilitated by formation of new hydrogen bonds by R153 and E157. Additional electrostatic interactions between H150 and K161 of α5 and D394 and E397 of α15 further assist in the movement of α5. Histidine residues have been implicated in SCARB2’s pH-dependent binding to β-GC (Zachos et al., 2012) and H150 is well positioned to act as a pH sensor for triggering conformational changes that regulate ligand binding.

In summary, helices α5 and α7 act as gatekeepers for enforcing a pH dependent opening and closing of the tunnel. Notably, although the tunnel is predominately hydrophobic, the top half is lined by a number of hydrophilic residues that are exposed to the solvent and is large enough to accommodate fatty acid molecules like sphingosine (Fig. S1C–E). A fatty acid-like molecule known as the “pocket factor” [presumed to be sphingosine in EV71 (Wang et al., 2012)] was shown to bind into a hydrophobic pocket beneath the canyon of EVs (Rossmann et al., 2002; Wang et al., 2012). Receptor binding at the canyon dislodges this viral “pocket factor” from the capsid protein and initiates uncoating (Ren et al., 2013; Zhang et al., 2008). Since EV71 uncoating occurs under acidic conditions, it is conceivable that acidic conditions first convert the lipid transfer tunnel of SCARB2 from a closed form as seen in nSCARB2 to an open form as observed in aSCARB2, permitting SCARB2 to suck the “pocket factor” from the viral capsid and thus initiate the uncoating process of EV71.

### SCARB2 dislodges “pocket factor” from EV71 virion

To prove that SCARB2 initiates uncoating of EV71 by removing the “pocket factor” from EV71 virions, we incubated mature virions with excessive [^3^H]-labeled sphingosine (Fig. S2). The natural lipid present in the capsid of the virions could be replaced by radio-labeled sphingosine in a time dependent manner. Next, we mixed mature virions containing this [^3^H]-labeled sphingosine with excess recombinant SCARB2-His-tag protein and Ni-NTA beads under acidic conditions (pH 5.0). The mixture was incubated at 37°C and aliquots were withdrawn at different time intervals for analysis. SCARB2 or SCARB2-EV71 complexes were removed from the samples by low-speed centrifugation. The Ni-NTA bead-bound His-tagged protein was sedimented upon centrifugation. The supernatant containing EV71 particles was ultracentrifuged to pellet EV71 particles. Radioactivity associated with EV71 particles was measured and the relative protein content of EV71 capsid was evaluated by Western blot.

**Figure 2:**
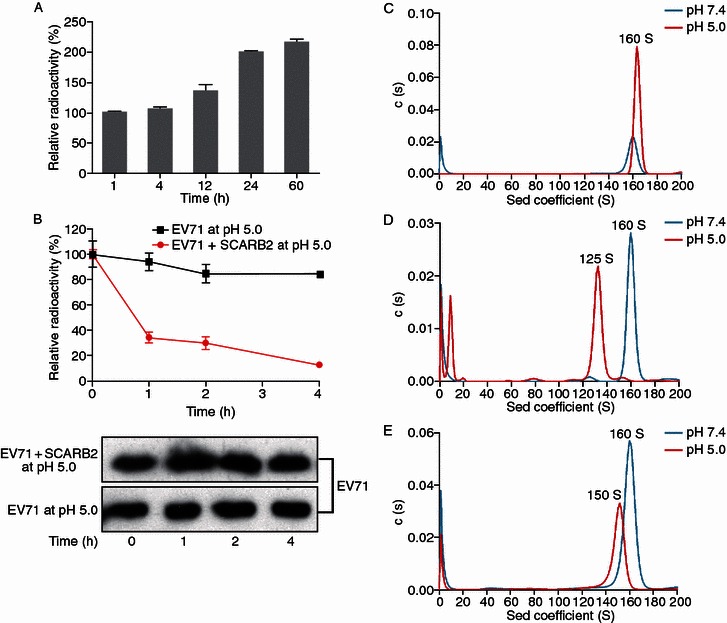
**SCARB2-mediated expulsion of lipid from EV71 virions triggers conformational change of EV71 mature virions under acidic conditions**. Competitive displacement of natural lipid from EV71 capsid proteins using [^3^H]-labeled sphingosine. (A) Time dependence of competitive [^3^H]-labeled sphingosine binding to EV71 mature virions. Purified EV71 mature virions were incubated with [^3^H]-labeled sphingosine (100 nmol/L) for the indicated times in PBS buffer (pH 7.4). EV71 virions were pelleted, washed for 3 times using PBS buffer (pH 7.4) and the radioactivity associated with them was estimated. Results shown are the mean ± SEM of three independent experiments. (B) Time dependence of [^3^H]-labeled sphingosine release from EV71 mature virions in the presence of SCARB2 under acidic conditions. EV71 mature virions bearing [^3^H]-labeled sphingosine were incubated with Ni-NTA beads in the absence or presence of an excess of recombinant SCARB2 (with a His tag) in acidic pH buffer (pH 5.0) at 37°C for the indicated times. Differential ultracentrifugation was used to pellet EV71 particles. Radioactivity and protein content was estimated. Results shown are the mean ± SEM of three independent experiments. The sedimentation coefficients of EV71 virions were measured by using analytical ultracentrifugation (AUC). (C) EV71 virions were suspended in neutral pH buffer (pH 7.4) or in acidic pH buffer (pH 5.0); (D) EV71 virions were incubated with SCARB2 under neutral condition (pH 7.4) or under acidic condition (pH 5.0) at 37°C for 1 h; (E) EV71 virions were pretreated using NLD compound, and then incubated with SCARB2 under neutral condition (pH 7.4) or under acidic condition (pH 5.0) at 37°C for 1 h. Assays carried out under neutral pH buffer (pH 7.4) and acidic condition (pH 5.0) are presented in blue and red curves, respectively. See also Fig. S2

Figure 2A shows it takes approximately 60 h for [^3^H]-labeled sphingosine to attain saturation in the capsid. However, under acidic conditions (pH 5.0), addition of recombinant SCARB2 to EV71 containing [^3^H]-labeled sphingosine followed by incubation of the mixture at 37°C for 1–4 h, resulted in a rapid decrease in [^3^H]-labeled sphingosine in virions when compared to the amount of radioactively labeled sphinghosine associated with similar amount of virions incubated under identical conditions but without SCARB2 (Fig. 2B). SCARB2 removes ~70% and 90% [^3^H]-labeled sphingosine from EV71 virions within 1 h and 4 h, respectively, while, EV71 alone loses almost no [^3^H]-labeled sphingosine at pH 5.0 solution buffer after 4 h incubation. These results demonstrate that SCARB2 can dislodge the “pocket factor” from the viral capsid at low pH.

### SCARB2 triggers uncoating of EV71 under low pH conditions

During the process of uncoating, the viral capsid undergoes large conformational changes, which can be monitored by sedimentation velocity experiments. Therefore, we performed analytical ultracentrifugation (AUC) studies on EV71 alone and in the presence of SCARB2. Mature virions sediment at ~160 S regardless of the pH (Fig. 2C). Under neutral conditions (pH 7.4), addition of recombinant SCARB2 to EV71 followed by incubation of the mixture at 37°C for 1 h, resulted in conversion of only ~5% of the virions into ~125 S particles (also known as A particles), that represent uncoating intermediates. In contrast to this, when the pH was lowered to 5.0 in the presence of SCARB2, almost all the mature virions were converted to ~125 S particles (Fig. 2D). Remarkably, a pretreatment of the mature virions with NLD (10 μg/mL), an EV71 “pocket factor” analog that functions as a stabilizer of EV71 virions (De Colibus et al., 2014) interfered with the uncoating process. Virions pre-treated with NLD and incubated with SCARB2 under acidic conditions (pH 5.0) at 37°C for 1 h exhibited a sedimentation co-efficient of ~150 S (Fig. 2E). Since the capsid of EV71 does not change morphology under neutral conditions, NLD binding had no effect at neutral pH (Fig. 2E). This indicates that conformational alternation of EV71 virions mediated by SCARB2 at acidic pH can be locked by NLD at an early stage. Thus uncoating of EV71 requires SCARB2, and SCARB2 can only induce uncoating under acidic conditions. Furthermore, SCARB2-mediated uncoating can be efficiently blocked by the “pocket factor” analog NLD.

### The binding interface between SCARB2 and EV71

**Figure 3:**
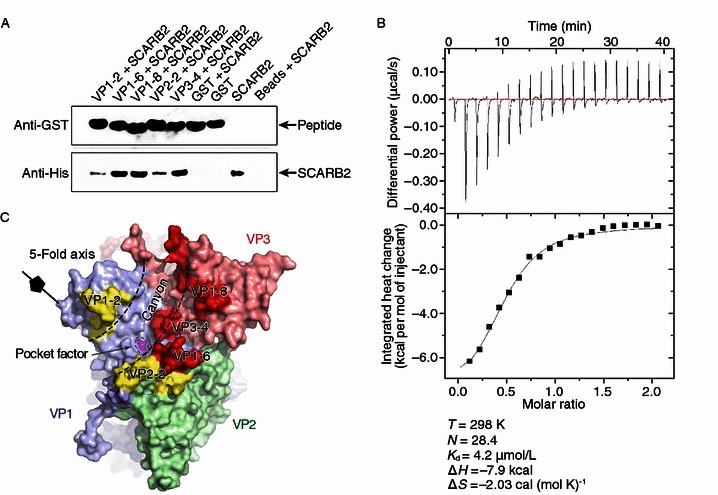
**Identification of binding interface between EV71 and SCARB2**. (A) GST pull-down assay for detecting interactions of SCARB2 with peptides located on the outer surface of the EV71 particle *in vitro*. Glutathione-Sepharose beads mixed with approximately 5 μg of GST-peptide were incubated with SCARB2. After the beads were washed, proteins that bound to the beads were analyzed by 15% SDS-PAGE, followed by Western blot analysis. The positions of peptide-GST and SCARB2 are marked on the right. (B) Titration of EV71 mature virions (7 μmol/L) with synthesized peptide of SCARB2 (aa 146–166, 400 μmol/L). Raw injection heats are shown in the top panel and the corresponding specific binding isotherm (calculated from the integrated injection heats and normalized to moles of injectant) are shown in the bottom panels. The derived dissociation constant (*K*_d_), stoichiometry parameter (*N*), and change in molar enthalpy (Δ*H*) and entropy (Δ*S*) are also shown. (C) Peptides around the “canyon” region of EV71 interact with SCARB2. Surface rendering of one icosahedral asymmetric unit (PDB code: 3VBH) of EV71. EV71 capsid protein VP1, VP2 and VP3 are colored in light blue, pale green and salmon, respectively. Peptides showing stronger interaction with SCARB2 are colored in red and peptides having a weaker affinity for SCARB2 in yellow. A 5-fold axis is shown as a black line and the pocket factor (cyan) indicated by a black arrow. See also Fig. S3 and Table S2

SCARB2, located on the cell membrane, interacts with EV71 to mediate its attachment. Although the binding sites of PSGL-1 and heparan sulfate on EV71 particles have been experimentally identified (Nishimura et al., 2013; Tan et al., 2013), the nature of the amino acids interacting with SCARB2 remains unclear. To map the location of SCARB2 binding on EV71, we investigated the virus-receptor interface using GST pull-down assays. We systematically selected 18 peptides located on the outer surface of the EV71 particle (Table S2), including the VP1 BC loop, VP1 GH loop, VP2 EF loop and VP3 GH loop. Next, these peptides fused with a GST tag were expressed in *E. coli* and tested for their ability to bind SCARB2 using a GST pull-down assay. The results showed that only peptides VP1-2, VP1-6, VP1-8, VP2-2 and VP3-4 could bind to recombinant SCARB2 (Fig. 3A and data not shown). Peptides VP1-6, VP1-8 and VP3-4 showed the most potent interaction, while peptides VP1-2 and VP2-2 interacted more weakly (Fig. 3A). The pull-down results show that the canyon region on the EV71 surface interacts with SCARB2 (Fig. 3C).

In addition, we demonstrated by isothermal titration calorimetry (ITC) that a peptide corresponding to residues 146–166 of SCARB2 interacted with EV71 mature virions *in vitro* (Fig. 3B). Residues 146–166 of SCARB2 cover a small part of α4, the α4, α5 linker, and the whole α5, exhibit variable conformations at different pHs (Fig. S3). Together with the observation that the G-H loops of VP1 and VP3 (peptides VP1-6 and VP3-4 respectively) alter their conformations during EV71 uncoating (Ren et al., 2013; Wang et al., 2012), these results indicate that EV71-SCARB2 complex undergo a series of conformational changes at the binding interface to trigger viral uncoating as pH decreasing.

### Glycosylation of SCARB2 plays a key role in receptor binding

**Figure 4:**
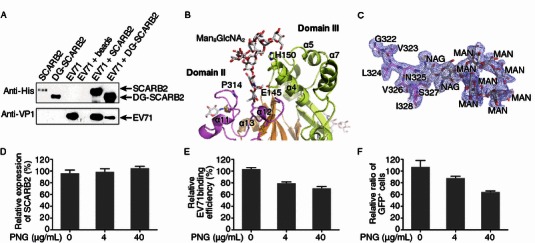
**Roles of glycosylation of SCARB2 in EV71 binding and infection**. (A) Pull-down assay for the interaction of SCARB2 or deglycosylated (DG) SCARB2 with EV71 mature virions *in vitro*. Ni-NTA beads mixed with approximately 3 μg of SCARB2 or deglycosylated SCARB2 were incubated with EV71 mature virions. Similar steps as Fig. 3A were carried out and the positions of SCARB2, DG-SCARB2 and EV71 are marked on the right. (B) Man_8_GlcNA_2_ at N325 extends to binding domain. The structure of Man_8_GlcNA_2_ and domain III are shown in the same format as in Fig. 1. (C) Electron density maps of Man_8_GlcNA_2_ at N325 of nSCARB2 (2*F*_*O*_ − *F*_*C*_ map contoured at 1.0 σ). EV71-GFP was used to infect 293A-hSCARB2 cell line pretreated with or without PNGase F. Fluorescence of GFP was determined 16 h post infection and EV71 infectivity was calculated and normalized to the infectivity to 293A-hSCARB2 cell line without any treatments, which was considered as 100%. (D) The levels of SCARB2 expression on the cell membrane from 293A-hSCARB2 cell line pretreated with or without PNGase F were monitored by flow cytometry assay. (E) Binding affinity comparisons of EV71 to 293A-hSCARB2 cell line pretreated with or without PNGase F. (F) Infection efficiency of EV71 to 293A-hSCARB2 cell line pretreated with or without PNGase F. Results shown are the mean ± SEM of three independent experiments for panel (D–F). See also Fig. S4

Glycosylation of functional receptors can play a critical role in the attachment of Picornaviruses to host cells (Fry et al., 1999; Vlasak et al., 2005). To verify the role of glycosylation of SCARB2 in EV71 infection, we tested the binding of EV71 to 293A-hSCARB2 cells pretreated with PNGase F using a flow cytometry based assay. PNGase F cleaves between the innermost GlcNAc moiety and asparagine residues from *N*-linked glycoproteins to remove glycans (Fig. S4A). 293A-hSCARB2 cells pretreated with PNGase F showed similar expression of SCARB2 on the cellular membrane when compared to the untreated control cells (Fig. 4D). However, PNGase F treated cells were impaired in their ability to bind EV71 (~30% reduction when compared to the untreated group) and the infection efficiency decreased by ~40% (Figs. 4E, 4F and S4C). We also tested the binding affinities between EV71 virions and SCARB2 decorated with different levels of glycosylations by performing *in vitro* pull-down assays. The results revealed that the binding of SCARB2 to EV71 decreased dramatically as the level of glycosylation went down (Fig. 4A). Taken together, these results suggest that glycosylation of SCARB2 may directly contribute to the attachment of EV71 to host cell.

Nine *N*-linked glycosylation sites with variable carbohydrate chain lengths from one to ten hexose units could be modeled into the structures of SCARB2. Amongst these multiple sites, based on the contour of the electron density and prior knowledge that lysosomal enzymes are often modified with Man_9_GlcNA_2_-P (Kim et al., 2009), the glycan at N325 of nSCARB2 was modeled as Man_8_GlcNA_2_ (Fig. 4C). Similarly, the glycan at N412 was modeled as Man_3_GlcNA_2_ (Fig. S4B). N325 is located at the bottom of a cleft formed between helices α11 and α12. Man_8_GlcNA_2_ protrudes to the surface out of this chasm and stations itself in vicinity of the cap. Residues from all three domains interact with this glycan. Notably, the sugar moieties adjacent to α4 and α5 are stabilized by P314, E145 and H150 (Fig. 4B). More importantly, the glycan is in proximity to EV71 binding sites of SCARB2 and the residues interacting with Man_8_GlcNA_2_ attached to N325 have been shown to be functionally associated with EV71 VP1 (Chen et al., 2012). This is consistent with the observation that the glycosylation of SCARB2 plays a role in viral attachment. Further, sialylated glycans (Yang et al., 2009), heparan sulfate glycosaminoglycan (Tan et al., 2013) and Suramin (Wang et al., 2013), which contain –SO_3_^-^ groups, bind EV71. Therefore these results suggest that Man_8_GlcNA_2_, with a negatively charged group, perhaps with other glycans, may facilitate attachment via ionic interactions with the EV71 surface.

## Discussion

### A complex model of SCARB2 binding to EV71

Viruses latch on to receptors using conserved residues (Rossmann et al., 2002). Members of the HEV-A sub-genus can be divided into two major groups based on their requirement of SCARB2 for infection, with EV71, CVA7, CVA14 and CVA16 using SCARB2 (Yamayoshi et al., 2012). Multiple sequence alignment of capsid proteins of all members belonging to HEV-A reveals that residues P96, L97, N102 of B-C loop, D219 of G-H loop, K285, S290 located at the C-terminus of VP1, G140, T141, E142, P147 of E-F loop of VP2 and H180, A181, R182, D183 of G-H loop of VP3 are highly conserved in all HEV-A viruses dependent on SCARB2 for infection (Fig. S5A). As expected, all these conserved residues lie within the five peptides that bound SCARB2 (Fig. 3A and 3C). Moreover, some of these residues have been previously reported to play essential roles in both attachment and uncoating (Ren et al., 2013; Wang et al., 2012).

**Figure 5:**
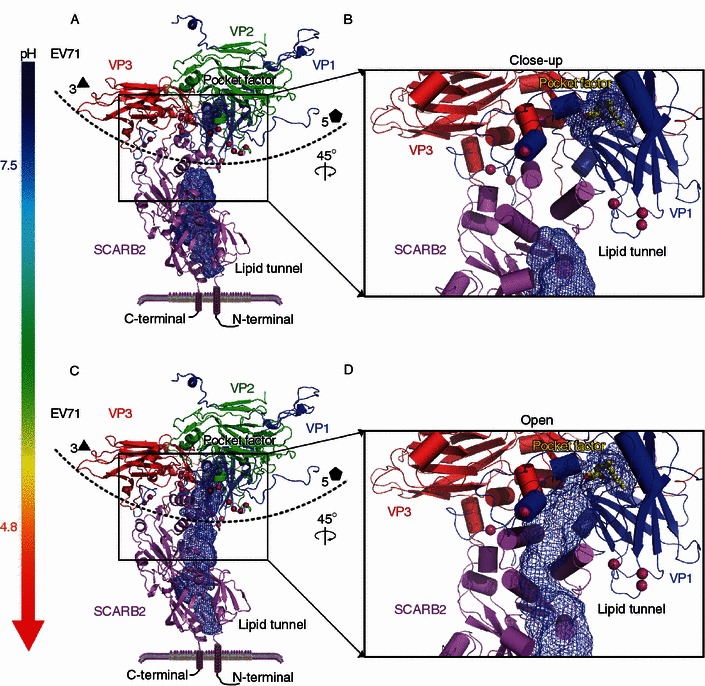
**Putative molecular mechanism of SCARB2-assisted attachment and uncoating of EV71**. The models of complexes of nSCARB2 (A), aSCARB2 (C) and one icosahedral asymmetric unit of EV71 (PDB code: 3VBH). VP1, VP2, VP3 and SCARB2 are drawn in blue, green, red and violet respectively. Potential residues involved in the binding of EV71 with SCARB2 are shown as spheres. The luminal tunnel of SCARB2 and hydrophobic pocket in VP1 from EV71 are represented as blue meshes. Pocket factor is shown in sticks. (B) and (D) are an enlarged representation of the EV71-SCARB2 interface. See also Figs. S5 and S6

To visualize the mode of binding of EV71 with nSCARB2 (Fig. 5A) and aSCARB2 (Fig. 5C), we constructed models of the virus-receptor complex using HEX8.0 (Mavridis et al., 2007). Two best models, with the lowest docking scores (docking score: −612 and −552 kJ/mol respectively), were selected from 25 docking conformations (See Extended Experimental Procedures). The results of the pull-down assay were used to evaluate if these models were reasonable (See Extended Experimental Procedures). The resulting complexes involve interactions between VP1-2, VP1-6, VP1-8 and VP3-4 of EV71 and the binding domain of SCARB2. In addition, the interaction surface between SCARB2 and EV71 as predicted by the model is consistent with the results of multiple sequence alignments. Furthermore, the complexes are quite stable, remaining intact after 10 ns of molecular dynamics simulation (Fig. S5B and S5C). According to these models of the EV71-SCARB2 complex, the hydrophobic pocket of the EV71 VP1 capsid protein lies adjacent to the lipid-transfer tunnel of SCARB2. In this plausible mode of binding, helices α4 and α5 located on the viral binding domain of SCARB2 are inserted into the canyon of EV71 capsid, and are flanked by helices α7 and α15 which sit towards the five-fold and three-fold axes of EV71, respectively. Such a configuration of the capsid with respect to SCARB2 positions conserved residues, including N102 of B-C loop, D219 of G-H, and K285 of the C-terminus of VP1 and R182, D183 of G-H loop of VP3 appropriately for interactions with domain III of SCARB2 (Fig. 5B and 5D).

### Model for SCARB2-mediated entry of EV71 into host cell

**Figure 6:**
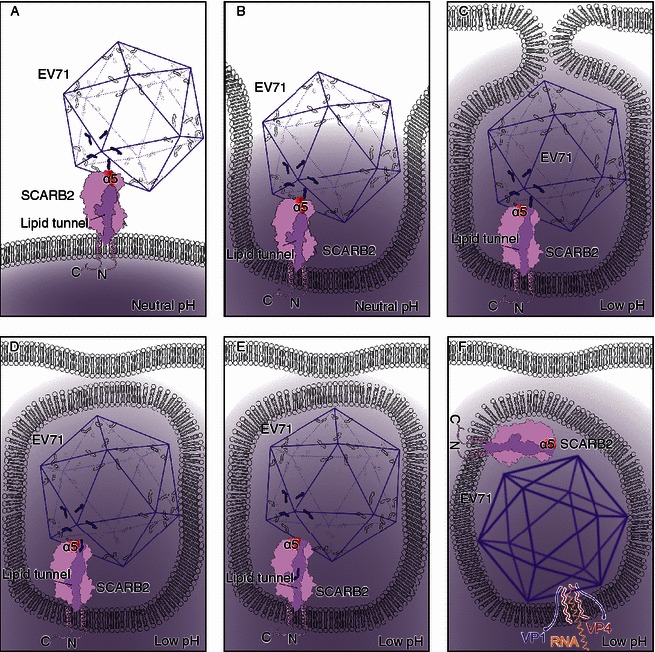
**Cartoon of SCARB2-mediated EV71 entry**. SCARB2 is inserted in the membrane with the ectodomain oriented towards the exofacial leaflet of the membrane. Pocket factor in VP1 is drawn as a “worm”. EV71 mature virion is shown as a purple icosahedron, and the deep-purple bigger icosahedron represents the EV71 uncoating intermediate. (A) EV71 in the process of attachment to the host cell membrane. (B) EV71 recognizes and interacts with cellular receptor SCARB2. (C) SCARB2 changes its conformation to open its “lipid-binding” tunnel at low pH value (<5.5) (upon internalization and transfer to the late endosome). (D) Expulsion of pocket factor from the viral capsid occurs. (E) Pocket factor is delivered to the membrane through the tunnel of SCARB2, meanwhile, EV71 undergoes a series of conformational changes as part of uncoating. (F) EV71 might dissociate from SCARB2 and form a channel in the membrane to release its RNA using the VP1 N-terminus and VP4

Mature EV71 virions harbor natural lipids, referred to as “pocket-factors”, within capsid proteins. Release of this lipid is a pre-requisite for the correct uncoating of EV71 (Grant et al., 1994; Smith et al., 1986). Under acidic conditions, such as encountered in the late endosome, parts of helices α4 and α5 of the viral binding domain of SCARB2 move away from helix α7, resulting in opening of the lipid-transfer tunnel. During viral attachment, the tunnel is positioned such that it lies adjacent to the hydrophobic pocket of VP1 (Fig. 5D). Conversely, under neutral conditions, α4 and α5 close the lipid-binding tunnel and block communication between the tunnel and the hydrophobic pocket of VP1 (Fig. 5B). A working model for the role of SCARB2 in the attachment and uncoating of EV71 can be built by combining results of our structure-function studies and previously published biochemical data (Fig. 6).

Under physiological conditions, EV71 virions recognize and attach to SCARB2 located on the cell membrane. Residues from the EV71 canyon region and the viral binding domain of SCARB2 in conjunction with glycans of SCARB2, form this critical interaction between the virus and the receptor (Fig. 6A and 6B). After attachment, the virions are internalized and transferred into endosomes. Under acidic conditions in the late endosomes, the amine “head” of sphingosine, a putative “pocket factor”, bears a positive charge, while α4, α5, α7 of SCARB2 and the late endosomal membrane are negatively charged (Fig. S6A and S6B). The pH triggered reorganization of helices α4, α5 of SCARB2 open, activate the lipid-transfer tunnel, and facilitate expulsion of viral “pocket factor” to trigger receptor-induced uncoating of the viral capsid (Fig. 6C and 6D). EV71 undergoes a series of conformational changes to first form an uncoating intermediate (Fig. 6E); followed by the N-terminus of VP1 and VP4 transiting from inside to outside, leading to the formation of a channel in the membrane through which the viral RNA is transported and released into the cytoplasm (Ren et al., 2013; Wang et al., 2012) (Fig. 6F).

## Conclusion

Our structure/functional analyses provides a series of crucial functional insights into the role of SCARB2 in cell entry by EV71 and several closely related type A human enteroviruses. Under neutral conditions, a cap closes the entrance of the lipid-transfer tunnel of SCARB2. When exposed to acid pH in the late endo/lyso-somes, the cap undergoes conformational changes that lead to the opening of the lipid-transfer tunnel. Upon opening of the lipid-transfer channel under acidic conditions of endo/lyso-somes, SCARB2 dislodges the “pocket factor” from the capsid of EV71 and translocates it through the tunnel in a manner analogous to lipids transported by CD36 and SR-BI. Removal of the “pocket factor” induces uncoating of EV71.

## Materials and Methods

### Crystallization

Materials, such as cells and plasmids, and methods for protein purification can be found in the Extended Experimental Procedures. Crystals were grown at 16°C using the hanging drop vapour diffusion method. 1 μL drops contained protein mixed with reservoir solution in 1:1 ratio. Crystals of nSCARB2 expressed in 293T cells and Sf9 cells were obtained from protein eluted in the first peak during ion exchange chromatography after 2–3 days in a condition containing 0.1 mol/L HEPES pH 7.5, 25% *w*/*v* PEG 3350 and 0.1 mol/L HEPES pH 7.5, 10% *v*/*v* 2-Propanol, 20% *w*/*v* Polyethylene glycol 4000 respectively. While crystals of aSCARB2 expressed in Sf9 cells were obtained in a condition containing 0.2 mol/L ammonium sulfate, 0.1 mol/L sodium acetate trihydrate pH 4.8, and 30% *w*/*v* polyethylene glycol monomethyl ether 2000.

### Structure determination

Diffraction data sets for nSCARB2 (expressed in 293T cells and Sf9 cells) and aSCARB2 were collected at beam line BL5A and BL17A of the Photon Factory (PF) synchrotron facility in Japan with the highest resolution being 3.0 Å, 2.8 Å and 3.65 Å, belonging to space groups of *C2*, *P2*_*1*_ and *P2*_*1*_ respectively. Data sets were processed and scaled using the HKL2000 package (Otwinowski and Minor, 1997). Data analysis and anisotropic processing can be found in the Extended Experimental Procedures. The initial structure solutions of nSCARB2 were obtained by molecular replacement using the program Phaser v2.1 (McCoy et al., 2007) with the crystal structure of SCARB2 (Protein Data Bank [PDB] entry: 4F7B (Neculai et al., 2013)) as a search template. Manual model building and refinement were performed using COOT (Emsley and Cowtan, 2004) and PHENIX (Adams et al., 2010) following rigid body refinement, energy minimization, B-factor refinement and group NCS constraints. The r.m.s. deviations between the two NCS-related subunits of nSCARB2 (expressed in 293T cells and Sf9 cells) are 0.029 Å and 0.037 Å respectively. The r.m.s. deviation between the four NCS-related molecules of aSCARB2 are 0.028 (chain A-B), 0.021 (chain A-C) and 0.029 Å (chain A-D) respectively. The structures of nSCARB2 expressed in 293T cells and Sf9 cells are almost identical with an r.m.s. deviation of 0.21 Å for all C α atom pairs and with an r.m.s. deviation of 0.32 Å for the C α atom pairs of the “cap”. Thus the structure of nSCARB2 (expressed in 293T cells) is selected as structural analysis to aSCARB2. Chain A from all analyses was selected for following structural analysis. Structural figures were drawn with the program PyMOL (DeLano, 2002).

### SCARB2-mediated dislodge of [^3^H]-labeled sphingosine from EV71 capsid under acidic condition

EV71 production and purification can be found in the Extend Experimental Procedures. Competitive displacement of natural lipid from EV71 capsid proteins using [^3^H]-labeled sphingosine. For each assay, 400 μL of purified EV71 mature virions (20 μg/mL) were incubated with 8 μL [^3^H]-labeled sphingosine (0.1 mCi/mL; 5 μmol/L; American Radiolabeled Chemicals, Inc.) at room temperature for the indicated times (1 h, 4 h, 12 h, 24 h and 60 h respectively) in PBS buffer (pH 7.4). Then EV71 virions were pelleted, washed for 3 times using PBS buffer (pH 7.4) and the radioactivity associated with them was measured. Radioactivity data were further normalized to the one of incubation for 1 h, which was considered as 100%. All reactions reported here were repeated triple times.

400 μL of purified EV71 mature virions (20 μg/mL) pretreated with 8 μL [^3^H]-labeled sphingosine (5 μmol/L) for 60 h were incubated with Ni-NTA beads in the absence or presence of mammalian expressed SCARB2 with an His tag at a molar ratio of 60:1 (SCARB2:Virus) in acidic pH buffer (pH 5.0) at 37°C for the indicated times (0 h, 1 h, 2 h and 4 h respectively). Centrifuged at 3000 rpm for 3 min to collect Ni-NTA beads, the supernatant was then used to pellet EV71 particles at 45,000 rpm for 1.5 h. The radioactivity of pelleted EV71 particles was determined, meanwhile, the relative protein contents were estimated by Western blot. Radioactivity data were further normalized to the one of the control group (in the absence of recombinant SCARB2 for 0 h), which was considered as 100%. All reactions reported here were repeated triple times.

### Analytical ultracentrifugation

EV71 full particles were mixed with mammalian expressed SCARB2 at a molar ratio of 1:60 at pH 7.4 and pH 5.0, respectively and then incubated at 37°C for 1 h. The full particles alone were analyzed under identical conditions as a control. NLD, an inhibitor of EV71 which can prevent viral uncoating was also used in this experiment with pre-incubation with EV71 full particles at a molar ratio of 240:1 (NLD:Virus) at room temperature for 4 h and then addition of SCARB2 as mentioned above. Sedimentation velocity experiments were performed on a Beckman XL-I analytical ultracentrifuge at 20°C. Different preprocessed virus samples were diluted with corresponding buffer (PBS pH 7.4 or Na-Citrate pH 5.0) to 400 μL at an A_280nm_ absorption of about 0.4. Samples were loaded into a conventional double-sector quartz cell and mounted in a Beckman four-hole An-60 Ti rotor. Data were collected at 12,000 rpm at a wavelength of 280 nm. Interference sedimentation coefficient distributions were calculated from the sedimentation velocity data using the SEDFIT software program (www.analyticalultracentrifugation.com).

### Role for glycosylation of SCARB2 in EV71 attachment and infection

293A-hSCARB2 cell line, which stably expressed human SCARB2 on the cell surface of 293A cells was established in our lab previously. EV71-GFP virus was prepared with enhanced green fluorescent protein (EGFP) gene inserted into EV71 genome between virus 5′ UTR and coding region such that GFP gene can be transcribed and translated during viral replication only. 293A-hSCARB2 cells were seeded in polylysine pretreated 96-well plate and cultured in complete DMEM. One day later, the cells were treated with 0, 4 or 40 μg/mL PNGase F at 37°C for 1 h. After washing with PBS, EV71-GFP virus was added to pretreated cells at 0.1 MOI. Sixteen hours post incubation, the cells were photographed under fluorescence microscopy, and then digested with trypsin and fixed with 4% paraformaldehyde (PFA). The GFP positive cells were analysed by flow cytometry, which can be found in the Extended Experimental Procedures. Deglycosylation of SCARB2 by PNGase F and the interaction between SCARB2 and EV71 *in vitro* by Pull-down assay can be found in the Extended Experimental Procedures.

### Molecular modelling and molecular dynamic simulation

Protein-protein docking was used to obtain an ensemble of complexes between deglycosylated aSCARB2 and capsid proteins (VP1–3) of EV71. The results of binding interface mapping provide directions for EV71-SCARB2 docking. More details can be found in the Extended Experimental Procedures.

### Isothermal titration calorimetry

Highly purified EV71 mature virions and the synthesized peptide (aa 146–166 of SCARB2) were in ITC buffer (PBS buffer pH 7.4). The SCARB2 peptide (aa 146–166, 400 μmol/L) was loaded into the syringe of the microcalorimeter (GE Company), with EV71 mature virions in the cell at a concentration of 7 μmol/L. Injections of 10 μL of the peptide were made into the cell at 25°C. Data were analysed by the Origin 7 software.

## Accession Codes

Coordinates and structure factors have been deposited with RCSB accession codes: 4TVZ, 4TW0, 4TW2.

## Electronic supplementary material

Below is the link to the electronic supplementary material.

Supplementary material 1 (PDF 1473 kb)

## Abbreviations

AUC: analytical ultracentrifugation
β-GC: β-glucocerebrosidase
CVA16: Coxsackievirus A16
EV71: Enterovirus 71
GD: Gaucher disease
HFMD: hand-foot-and-mouth disease
ITC: isothermal titration calorimetry
PSGL-1: P-selectin glycoprotein ligand-1
SCARB2: scavenger receptor class B 2
