# Supplementary material for: Molecular mechanism of SCARB2-mediated attachment and uncoating of EV71
Source: Protein Cell. 2014 Jul 2;5(9):692–703. doi: 10.1007/s13238-014-0087-3 (PMC4145081; doi:10.1007/s13238-014-0087-3)
Supplement: Supplementary file 1 — Supplementary material 1 (PDF 1473 kb) [file 13238_2014_87_MOESM1_ESM.pdf]

Supplementary information for: ***Molecular mechanism of SCARB2-mediated attachment and uncoating of EV71***

SUPPLEMENTARY FIGURES AND LEGENDS

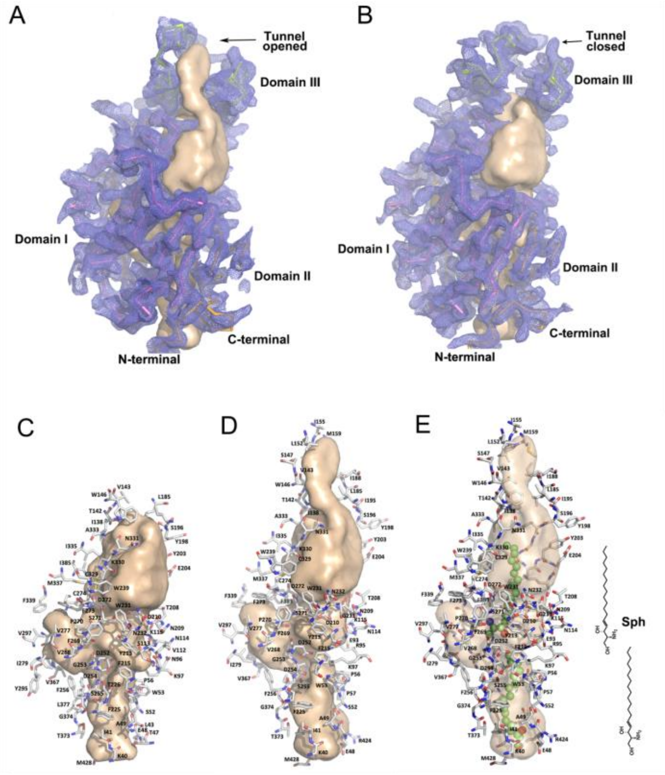

Figure S1: Two forms of the tunnel of SCARB2 and electron density maps of SCARB2 around the tunnel. Electron density maps of aSCARB2 (A) and nSCARB2 (B) around the tunnel ($2F_O$ - F_C map contoured at 1.0 σ). SCARB2 is shown as ribbon format and the cavity is represented as wheat surface. Domain I, Domain II and Domain III are colored in orange, violet and lemon, respectively. (C). Tunnel of nSCARB2; (D). Tunnel of aSCARB2. Side chains are numbered and shown in stick format. Oxygen and nitrogen atoms are colored red and blue, respectively. (E). Two sphingosine molecules (shown in green) docked into (D) and the cavity is shown as semi-transparent surface.

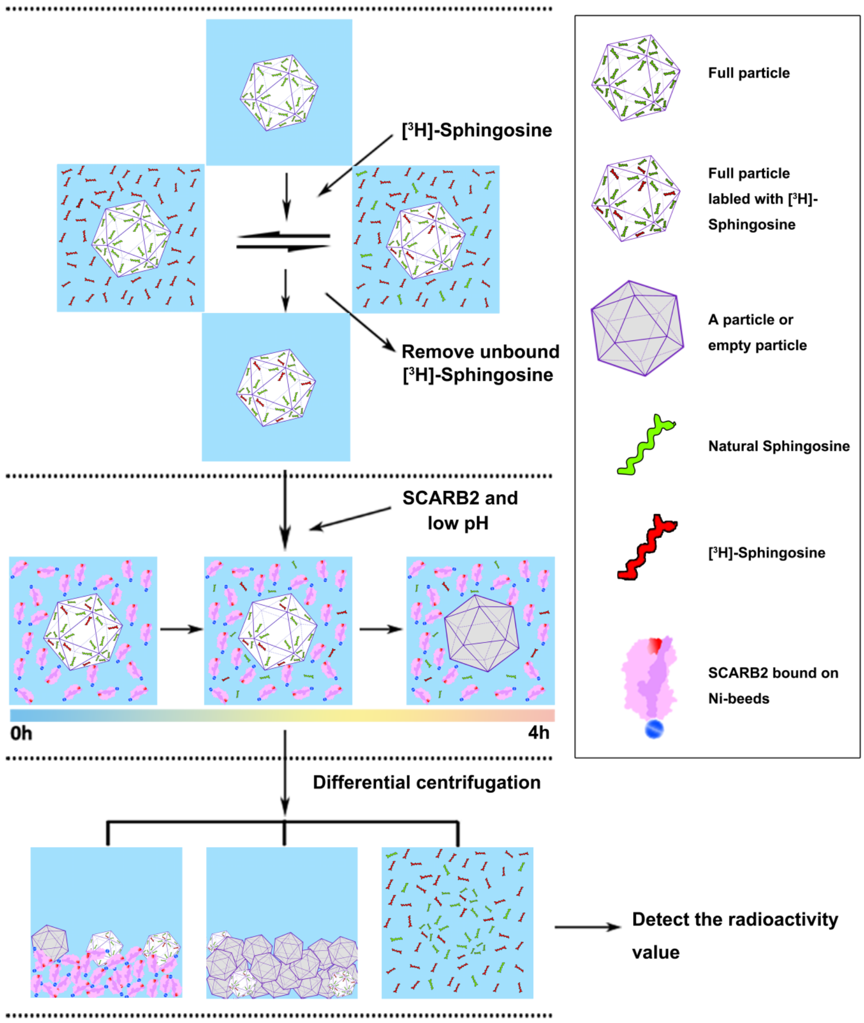

Figure S2: Schematic diagram depicting steps of assay for SCARB2 mediated lipid expulsion from EV71 virions. Step 1: Competitive displacement of natural lipid from EV71 capsid proteins using [3H]-labeled sphingosine; **Step 2:** Time-course of interaction of SCARB2 with EV71 virions harboring [3H]-labeled sphingosine under pH 5.0 condition at 37 ˚C; **Step 3:** Determination of the ability of SCARB2 mediated [3H]-labeled sphingosine release from EV71 virions by detecting residual radioactivity associated with EV71 particles and estimating EV71 capsid protein content.

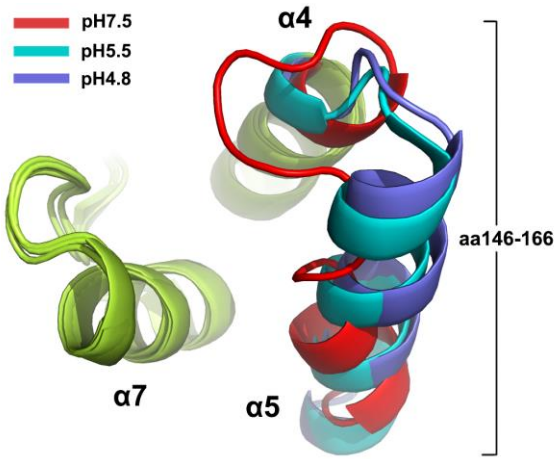

Figure S3: Structural comparisons of viral binding domain of SCARB2 at different pHs. Ribbon diagram showing the structural differences of the Domain III helical bundle at pH7.5, pH5.5 [PDB Code: 4F7B (Neculai et al., 2013), chain A] and pH4.8. The regions of the residue 146-166 of SCARB2 (pH7.5, pH5.5 and pH4.8) are presented in red, cyans and slate respectively.

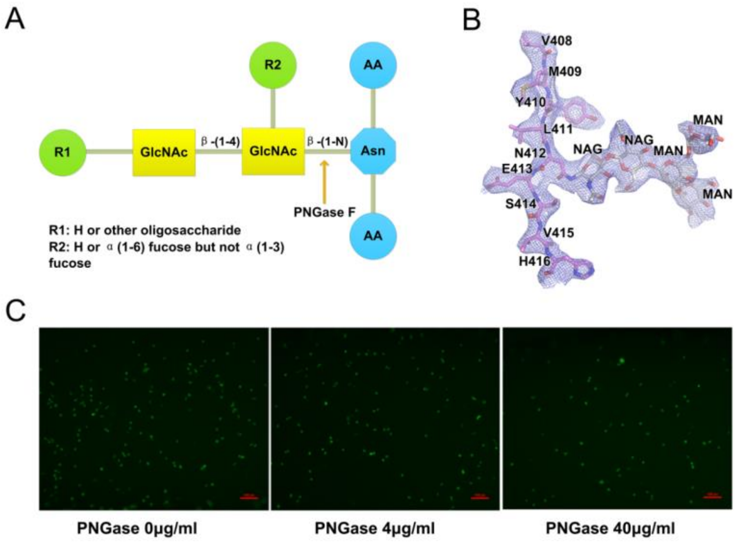

Figure S4: Roles of the glycosylation of SCARB2 in EV71 attachment and infection. (A). Schematic diagram showing site of digestion of PNGase F. The enzyme can cleave between the innermost GlcNAc and asparagine residues of high mannose, hybrid, and complex oligosaccharides from N-linked glycoproteins. Electron density maps of Man_3GlcNA_2 at N412 of nSCARB2 (B) ($2F_O$ - F_C map contoured at 1.0 σ). (C). Phenotype screening identified concentration-dependent reduction of EV71 proliferation following treatment with PNGase. 293A-hSCARB2 cells with or without pre-treatment by various concentrations of PNGase were infected with EV71-GFP virus at the MOI of 1 for 16h. EGFP was used to monitor viral infection.

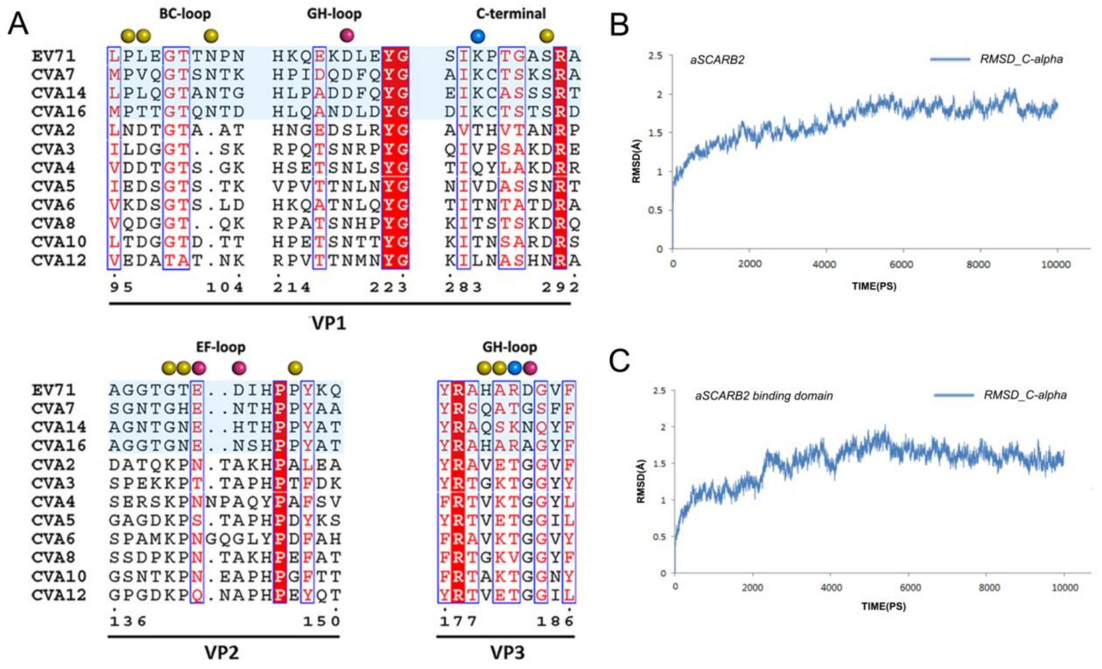

Figure S5: Analysis of EV71-SCARB2 complex binding mode. (A). Multiple sequence alignment of VP1, VP2 and VP3 of human enteroviruses A species (HEV-A). EV71, CVA7, CVA14 and CVA16 (colored in light blue background) which belong to the SCARB2-dependent infection group. The remaining viruses are SCARB2-independent. Residues labeled by a sphere are highly conserved in SCARB2-dependent infection group and are significantly distinct from corresponding residues belonging to the members of the other group. The red sphere and blue sphere represent negatively and positively charged residues respectively. The stability of aSCARB2-EV71 complex during molecular dynamic simulation. The SCARB2-EV71 complex was refined using molecular dynamic simulation with software Amber 12 (Götz et al., 2012). Variation of RMSD for aSCARB2 (B) and aSCARB2 binding domain (C) Cα atoms during the simulation. The system reaches equilibrium in 5 ns. The 10ns MD simulation showed that the complex was quite stable, despite the fact that the interaction between the two components was not restrained (backbone RMSD to the docking conformation was below 2 Å).

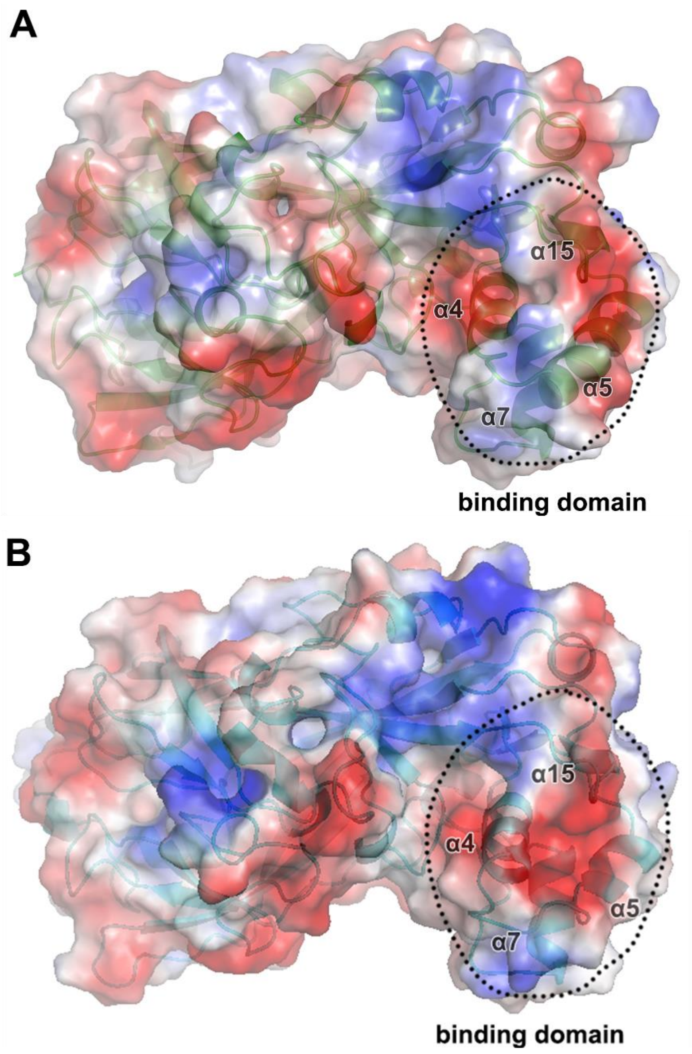

Figure S6: Surface electrostatic potential representation of SCARB2. Electrostatic surfaces of aSCARB2 (A) and nSCARB2 (B) contoured at ±3 kT/e. Negatively charged surface is shown in red; positively charged surface in blue. The EV71 binding domain of SCARB2 is marked by a black dotted line.

Table S1: Data collection and refinement statistics. Related to Figure 1

Name	nSCARB2(pH7.5)		aSCARB2(pH4.8)
	293T cell	sf9 cell	
**Data collection**			
Resolution (Å)	50.00-3.0 (3.11-3.00)	50.00-2.80(2.90-2.80)	50.00-3.65(3.71-3.65)
Unique reflections	24297 (2370)	28574(2674)	28909 (2941)
Space group	*C*2	*P21*	*P*21
Cell dimensions			
a (Å)	94.9	57.8	104.0
b (Å)	63.7	100.3	99.6
c (Å)	219.9	105.2	125.8
α (°)	90.00	90.00	90.00
β (°)	99.75	98.69	90.02
γ (°)	90.00	90.00	90.00
Redundancy	3.4(3.4)	3.7(3.4)	2.9 (2.9)
Completeness (%)	92.7 (91.6)	99.1(93.1)	98.4 (98.1)
R_{merge}	0.131 (0.637)	0.105(0.535)	0.141 (0.707)
$I/\sigma(I)$	8.87 (2.17)	9.98(1.8)	7.46 (2.05)
**Refinement**			
Resolution(Å)	3.00	2.80	3.65
No.reflections	18, 193	25398	23, 039
R_{work}/R_{free}	0.237/0.292	0.234/0.281	0.239/0.288
No. of non-H atoms			
Protein	6228	6156	12143
Glycans	720	496	992
Mean B-factor(Å^2)	31.2	46.5	69.1
**Ramachandran statistics (%)**			
Most favored	97.3	94.5	95.1
Allowed	2.7	5.5	4.9
Outliers	0	0	0
**R.m.s.deviations**			
Bond lengths(Å)	0.006	0.010	0.007
Bond angles(°)	1.244	1.434	1.302

Values in parentheses are for highest-resolution shell.
[a]$R_{merge} = \Sigma_{hkl} \Sigma_i |I(hkl)_i - \langle I(hkl)\rangle| / \Sigma_{hkl} \Sigma_i I(hkl)_i$,
[b]$R_{work} = \Sigma_{hkl} |Fo(hkl) - Fc(hkl)| / \Sigma_{hkl} Fo(hkl)$.
[c]R_{free} was calculated for a test set of reflections (5%) omitted from the refinement.

Table S2: Peptides of EV71 and peptide of SCARB2 used in this study for EV71-SCARB2 binding interface mapping. Related to Figure 3

Peptides	Sequence
VP1-1(88-102)	RAGLVGEIDLPLKGT
VP1-2(92-106)	EIDLPLEGTTNPNGY
VP1-3(98-112)	EGTTNPNGYANWDID
VP1-4(104-118)	NGYANWDIDITGYAQ
VP1-5(158-173)	PGAPKPDSRESLAWQT
VP1-6(212-225)	GEHKQEKDLEYGAC
VP1-7(236-250)	RTVGTSKSKYPLVVR
VP1-8(280-293)	GNSIKPTGASRA
VP2-1(65-78)	TLDTKLWEKSSKGW
VP2-2(136-150)	AGGTGTEDIHPPYKQ
VP2-3(153-167)	PGADGFELQHPYVLD
VP2-4(221-235)	PLDYDQGATPVIPIT
VP3-1(56-71)	NNVPTNATSLMERLRF
VP3-2(67-82)	ERLRFPVSAQAGKGEL
VP3-3(139-153)	GGPLPKDRATAMLGT
VP3-4(175-188)	THYRAHARDGVFDY
VP3-5(199-213)	QTNYVVPIGAPNTAY
VP3-6(230-242)	KDASDILQTGTIQ
SCARB2(146-166)	WSQVHFLREIIEAMLKAYQQK

EXTENDED EXPERIMENTAL PROCEDURES

Cells and plasmids

Human embryonic kidney (HEK) 293T cells were grown adherently in roller bottles at 37 ℃ in DMEM supplemented with 2% (v/v) fetal bovine serum (FBS) (Hyclone). Sf9 cells were cultured in Serum Free Medium at 27 ℃ on a shaker maintained shaking at 100 rpm. A series of constructs of the ectodomain of human SCARB2 were designed and inserted into the pHL_Sec vector for transformation into 293T cells. PHL_Sec vector is a secreted-expression vector used in mammalian expression system where a recombinant protein can be expressed with a chicken bactin tag at N-terminus and a 6x his-tag at the C-terminus. The constructs were parallel cloned into pFastBac_Bee vector for transformation into Sf9 cells. pFastBac_Bee is vector that has been modified from pFastBac-one and can express recombinant protein with a melittin tag at the N-terminus and a 6x his-tag at the C-terminus. Positive clones were identified by PCR and DNA sequencing and were transformed into DH5α Escherichia coli DH5α cells to obtain large amounts of recombinant plasmid DNA.

Protein expression and purification

293T cells were transfected with recombinant plasmid DNA using a PEI-mediated transfection protocol as described previously (Godbey et al., 2000). Cells were cultured at 37 ℃ for ~ 4-5 days until they began to detach. At this point, the medium was collected and processed for recovery of the secreted recombinant protein. Similarly, recombinant protein was produced using Sf9 cells by infecting 1 lit of cells (2 x 10^6/ml confluence) with 10 ml of P3 viral stock that was obtained following manufacturer's instructions (Invitrogen). The medium containing the

secreted protein was collected after 3–4 days of incubation.

The cell culture medium of 293T cells or Sf9 cells was harvested and centrifuged for 30min at 4000g. The supernatant was filtered through a 0.22 μm filter and then dialysed against 10-fold excess of PBS (pH=7.4). This procedure was repeated twice. The sample was then mixed with pre-equilibrated Ni-NTA Agarose (Qiagen) beads at a ratio of 5 ml settled beads per liter culture and stirred at 4 ℃ for 2h. The slurry was loaded onto a 15 ml gravity column (Bio-Rad) and washed with a buffer containing 20mM Hepes, pH 7.5, 50mM NaCl and 10% v/v glycerol for 20~30 column volumes. The beads were further washed by using a buffer containing 10 mM imidazole until no traces of the protein were detected in the flow-through. SCARB2 bound to the column was eluted in a buffer containing 50 mM imidazole, concentrated (Amicon Ultra-15 30,000 MWCO, Millipore) to approximate 500 μl and loaded onto a Superdex G200 gel filtration column (GE Healthcare) equilibrated with wash buffer. Fractions containing the protein were collected, pooled and further purified by ion exchange chromatography using a Resource Q column (GE Healthcare). Multiple peaks were observed during elution. Fractions for each peak were collected separately, concentrated to 5mg/ml and screened for crystallization.

Protein yields of different constructs were estimated by western blot (data not shown) and well-behaved ones such as construct-1(27-431), construct-2(28-431), construct-3(36-431), and construct-4(60-431) were chosen for large scale expression. Both oligomeric and monomer forms of SCARB2 were observed by size-exclusion chromatography, when expressed in Sf9 cells or in 293T cells. Construct-2(28-431) was selected for crystallization and subsequent biochemical assays. SCARB2 has ten potential *N*-glycosylated sites, containing a ~400 amino acid ectodomain and two transmembrane domains with a ~85kD molecular weight (Holmes, 2012).

Diffraction data analysis and anisotropic processing

The aSCARB2 diffraction pattern is close to being orthorhombic (*$P2_12_12$*), however scaling the integrated intensities in the 50-10 Å resolution range gave a Chi**2 of 2.08 when assuming twofold symmetry axes along a*, b*, c*, in contrast the Chi**2 was 1.04 when assuming a twofold symmetry axis along c* (*$P2_1$*) with the same error model. We therefore treated aSCARB2 as monoclinic. Two, two and four protein molecules in an asymmetric unit with a solvent content of 52%, 55% and 59% (corresponding to a Matthews coefficient V_M= 2.55, 2.72 and 2.96 $Å^3$ Da^{-1} (Matthews, 1968)) were found from crystals of nSCARB2 (expressed in 293T cells and Sf9 cells) and aSCARB2 respectively. Due to the anisotropic diffraction of nSCARB2 (expressed in 293T cells) and aSCARB2 crystals, ellipsoidal truncation and anisotropic scaling was applied using the online Diffraction Anisotropy Server (Strong et al., 2006). For nSCARB2 (expressed in 293T cells) the diffraction limits were 3.0 Å in the a* and b* directions, but only 3.7 Å along the c* direction, whilst for aSCARB2 the diffraction limits were 3.6 Å along c*, 3.7 Å along b*, only 4.3 Å in the a* direction. For nSCARB2, 24,297 reflections were in the initial data set, 6,078 were discarded, because they fell outside the specified ellipsoid with dimensions 1/3.0, 1/3.0, 1/3.7 Å along a*, b*, c* respectively. As to aSCARB2, 5,728 of the 28,892 reflections in initial data set were discarded for being outside the specified ellipsoid. Anisotropic scale factors were then applied to remove the anisotropy from the data sets. Lastly sharpening B factors of -61 $Å^2$ and -71$Å^2$ for nSCARB2 (expressed in 293T cells) and aSCARB2 respectively were applied to restore the magnitude of the higher resolution reflections diminished by anisotropic scaling. These anisotropically scaled data then were used for structure determination and model refinement.

Flow cytometry analysis

Cells were washed with PBS once, digested with 0.025 % typsin-EDTA solution for 5 min. Cells were then spun down at 3000 r/min, re-suspended and fixed with 4%

paraformaldehyde (PFA). The GFP positive cells were analysed by flow cytometry (BD calibur). The infection efficiency of EV71-GFP virus is presented as percentage of GFP positive cells.

EV71 production

EV71 (genotype C4), isolated from Fuyang, Anhui Province, China, was used to infect Vero cells at an MOI of 0.5 and the cells were cultured in Dulbecco's modified Eagle's medium (DMEM; Sigma) supplemented with 0.5% fetal bovine serum (FBS). The virus was collected 5–6 d post infection, centrifuged to remove cell debris, ultrafiltered and subjected to gel filtration. EV71 was purified as described previously (Wang et al., 2012).

PNGase F digestion

100μg of total mammalian expressed SCARB2 were incubated with 2.5μL of PNGase F (New England Bio Labs) for 1 hour at 37 ℃, following the manufacturer's instructions.

Pull-down assay

Mammalian expressed SCARB2 exhibits higher levels of glycosylation than SCARB2 expressed in insect cells, as judged by migration of protein bands on SDS-PAGE. Mammalian expressed SCARB2 and deglycosylated SCARB2 were used as samples to explore the roles of glycoslytion in EV71 binding. 3μg of SCARB2 with His-tag was incubated with 5μg purified EV71-Full at 4 ℃ for 1h in the presence of Ni-NTA beads (Qiagen). The beads were collected by centrifuging the mixture for 5 min at 13300 rpm, and subsequently washed 4 times with cold PBS to remove unbound EV71-Full. Then the samples were eluted with a small amount of

1M imidazole and the bound viruses were detected using EV71 specific antibody by western blot.

GST pull-down assay for detecting interactions of SCARB2 with peptides located at the outer surface of EV71 particle *in vitro*. Glutathione-Sepharose beads bound approximately 5 μg of GST-peptide were incubated with 5 μg mammalian expressed SCARB2 for 1h. After the beads were washed 4 times with cold PBS (pH 7.4), proteins were eluted with 10mM reduced glutathione and analyzed by western blots.

Western blot

The protein samples were subjected to 15% SDS-polyacrylamide gel electrophoresis, transferred onto a PVDF membrane and blocked by 5% skimmed milk in PBST, then incubated with 1: 2000 diluted anti-EV71 antibody (ab36367, Abcam), or 1:5000 diluted anti-His tag antibody. Extensive washing with PBST was followed by incubation with horse radish peroxidase (HRP) conjugated secondary antibody at a dilution of 1:10000. Western Luminescent Detection Kit (Vigorous) was used and the reaction was detected on X ray film (Fuji).

Molecular modeling and molecular dynamic simulation

Protein-protein docking software Hex 8.0 (Ghoorah et al., 2013; Macindoe et al., 2010; Ritchie et al., 2008; Ritchie and Venkatraman, 2010) was used to obtain an ensemble of complexes between deglycosylated aSCARB2 and capsid proteins (VP1~3) of EV71. 25 complexes with the lowest docking score were saved for further refinement. The two best docking clusters (docking score: -612 and -552 kJ/mol respectively) were selected among 25 docking conformations. These two docking clusters were evaluated by the results of the pull-down experiments: The first cluster have interactions with VP1-2, VP1-8 and VP2-2; the second cluster have interactions with VP1-2, VP1-6, VP1-8 and VP3-4. In the pull-down experiments, VP1-6, VP1-8

and VP3-4 show notable interaction with SCARB2. Thus, we selected the second docking cluster as an experiments-based docking conformation. The complexes were then refined using 10 ns molecular dynamic simulation with software Amber 12 (Götz et al., 2012). At first, Complexes were minimized with 1000 steps of steepest descent minimization and followed by 1000 steps of conjugate gradient minimization. Then, the whole system was heated to 300K in 10ps with 50kcal/mol restrain force on backbone of proteins. After a 1ns density equilibration, a 9ns production equilibration was performed with 20kcal/mol restrain force on backbone of VP1~3 (considering VP1, 2 and 3 constitute only part of the EV71 shell, it would be unreasonable to simulate without restraints). The SCARB2 was free to move in the production equilibration. For the ensemble of complexes between nSCARB2 and capsid proteins of EV71, nSCARB2 was superimposed onto aSCARB2-EV71 complex model.

Supplementary Reference

Götz, A.W., Williamson, M.J., Xu, D., Poole, D., Le Grand, S., and Walker, R.C. (2012). Routine microsecond molecular dynamics simulations with AMBER on GPUs. 1. Generalized born. Journal of Chemical Theory and Computation *8*, 1542.

Ghoorah, A.W., Devignes, M.D., Smaïl‐Tabbone, M., and Ritchie, D.W. (2013). Protein docking using case‐based reasoning. Proteins: Structure, Function, and Bioinformatics *81*, 2150-2158.

Godbey, W., Barry, M.A., Saggau, P., Wu, K.K., and Mikos, A.G. (2000). Poly (ethylenimine)-mediated transfection: a new paradigm for gene delivery. Journal of biomedical materials research *51*, 321-328.

Holmes, R.S. (2012). Vertebrate scavenger receptor class B member 2 (SCARB2): comparative studies of a major lysosomal membrane glycoprotein. Journal of Molecular Biochemistry *1*.

Macindoe, G., Mavridis, L., Venkatraman, V., Devignes, M.-D., and Ritchie, D.W. (2010). HexServer: an FFT-based protein docking server powered by graphics processors. Nucleic acids research *38*, W445-W449.

Matthews, B.W. (1968). Solvent content of protein crystals. Journal of molecular biology *33*, 491-497.

Neculai, D., Schwake, M., Ravichandran, M., Zunke, F., Collins, R.F., Peters, J., Neculai, M., Plumb, J., Loppnau, P., and Pizarro, J.C. (2013). Structure of LIMP-2 provides functional insights with implications for SR-BI and CD36. Nature.

Ritchie, D.W., Kozakov, D., and Vajda, S. (2008). Accelerating and focusing protein–protein docking

correlations using multi-dimensional rotational FFT generating functions. Bioinformatics *24*, 1865-1873.

Ritchie, D.W., and Venkatraman, V. (2010). Ultra-fast FFT protein docking on graphics processors. Bioinformatics *26*, 2398-2405.

Strong, M., Sawaya, M.R., Wang, S., Phillips, M., Cascio, D., and Eisenberg, D. (2006). Toward the structural genomics of complexes: crystal structure of a PE/PPE protein complex from Mycobacterium tuberculosis. Proceedings of the National Academy of Sciences *103*, 8060-8065.

Wang, X., Peng, W., Ren, J., Hu, Z., Xu, J., Lou, Z., Li, X., Yin, W., Shen, X., and Porta, C. (2012). A sensor-adaptor mechanism for enterovirus uncoating from structures of EV71. Nature Structural & Molecular Biology *19*, 424-429.
